# Growth performance, immune status, gastrointestinal tract ecology, and function in nursery pigs fed enzymatically treated yeast without or with pharmacological levels of zinc

**DOI:** 10.1093/jas/skac094

**Published:** 2022-03-22

**Authors:** Brenda Christensen, Cuilan Zhu, Mohsen Mohammadigheisar, Hagen Schulze, Lee-Anne Huber, Elijah G Kiarie

**Affiliations:** 1 Department of Animal Biosciences, University of Guelph, Guelph, Ontario, N1G 2W1, Canada; 2 Livalta, AB Agri Ltd., Peterborough, Cambridgeshire, PE2 6FL, UK

**Keywords:** enzymatically treated yeast, growth performance, immunocompetence, gut physiology, piglets, zinc oxide

## Abstract

Growth performance and physiological responses of nursery piglets when fed enzymatically treated yeast (HY40) and pharmacological ZnO alone or in combination were investigated. A total of 144 pigs (21 d old, BW 7.32 ± 0.55 kg) were placed in 36 pens (4 pigs/pen). Pigs were randomly assigned to one of four dietary treatments (*n* = 9): 1) control corn-wheat-soybean meal diet (control), 2) control + HY40 (HY40), 3) control + (ZnO) and 4) control + HY40 + ZnO (HY40+ZnO). Inclusion of HY40 and ZnO was 0.5% and 3,000 ppm in phase I (days 0 to 14), respectively, and halved in phase II (days 15 to 42). All diets contained 0.2% TiO_2_ for determination of apparent total tract digestibility (ATTD) of components. Body weight and feed disappearance were recorded weekly. One pig per pen was killed for organ weights, blood, and tissue samples on day 14. Except for phase II, when HY40 + ZnO pigs had greater average daily feed intake (*P* = 0.004) than all other treatments, there were no (*P* > 0.05) interactions between HY40 and ZnO on growth performance. Pigs fed HY40 or ZnO containing diets were heavier (*P* < 0.05) than pigs fed without by the end of the study. On day 14, pigs fed additives exhibited higher (*P* ≤ 0.009) ATTD of dry matter (DM) and gross energy (GE) than control pigs. On day 28, pigs fed control, HY40, and HY40 + ZnO had greater (*P* ≤ 0.022) ATTD of DM, crude protein, and GE than piglets fed ZnO only. Pigs fed HY40 + ZnO had lower ileal digesta *Escherichia coli* concentration (*P* < 0.05) than HY40 and control pigs. Ileal digesta of pigs fed ZnO diets had higher *lactobacillus* to *E. coli* ratio (1.44 vs. 1.20; *P* = 0.001), exhibited higher concentrations of acetic (*P* = 0.01) and butyric acid (*P* = 0.01) but lower lactic (*P* = 0.02) and total short chain fatty acids (*P* = 0.033) than pigs fed non-ZnO diets. Greater (*P* < 0.05) mRNA expression of nutrient transporters, tight junction proteins, and fecal excretion of zinc (Zn) was observed in ZnO pigs relative to non-ZnO pigs. Pigs fed HY40 diets had greater (*P* = 0.002) villus height to crypt depth ratio (VH:CD) than non-HY40 pigs. The concentration of plasma IgA was higher (*P* = 0.04) in HY40 + ZnO pigs relative to other pigs, whereas HY40 pigs showed higher *(P* < 0.001) jejunal IgA than non-HY40 pigs. Although the mode of action of HY40 and ZnO differed, the present study indicated that HY40 improved growth performance and jejunal function and immunity, making HY40 an effective alternative to pharmacological ZnO in nursery pigs feeding programs.

## Lay Summary

Abrupt weaning of pig results in nutritional, environmental, and psychological stresses leading to reduced feed intake, little or no weight gain and in some instances diarrhea, morbidity, and mortality. The post-weaning growth performance lag is often managed by use of antimicrobial growth promoters (AGP), pharmacological levels of trace elements such as zinc oxide (ZnO), specialty feed ingredients, and additives. However, many jurisdictions have already banned, and others are considering banning/restricting use of AGP to abate burgeoning levels of antimicrobial resistance in pathogens of animal and human. The European Union confirmed banning pharmacological ZnO effective June 2022 because of environmental pollution. In this context, identifying alternative nutritional strategies for managing newly weaned pigs is of global concern. Yeast derivatives have been proposed to be beneficial in transitioning piglets. However, there is little available information on comparative efficacy of yeast derivatives and pharmacological ZnO. The current study demonstrated that enzymatically treated yeast (HY40) had positive effects on growth, intestinal histomorphology, nutrient digestibility, and immune indices in nursery pigs. Through differing modes of action, overall growth performance of HY40 piglets was comparable to that of pharmacological ZnO piglets.

## Teaser

Although the mode of action of HY40 and ZnO differs, piglets fed HY40 had commensurate growth, and indices for gastrointestinal function and health, indicating that HY40 is an effective alternative to pharmacological ZnO for nursery pigs.

## Introduction

The weaning process results in pigs being exposed to nutritional, environmental, and psychological stress that leads to reduced feed intake and weight gain, and increased morbidity and mortality. Low feed intake and diarrhea leads to thinning of the intestinal mucosal layer and suppressed immune system in addition to reduced growth performance (Pluske, 2016). Sub-therapeutic levels of in-feed antibiotics (AGP) and speciality feed ingredients and additives are often used in nursery diets to minimize this growth lag (Kiarie et al., 2016; Pluske, 2016). However, there are growing concerns in Canada and around the world regarding indiscriminate use of antibiotics and linkage to the emergence of antibiotic resistant pathogens (Ciesinski et al., 2018). Moreover, following the ban of use of antimicrobial growth promoters in the European Union, pharmacological zinc oxide (ZnO) has been commonly used to prevent and control post-weaning diarrhea (PWD) and bowel edema disease in young pigs (Eriksen et al., 2021). The European Commission confirmed the EU-wide ban on the use of ZnO at pharmacological levels (>150 ppm) in piglet feed, effective June 2022 due to excess zinc excretion (EMA, 2017). Along with environmental implications of ZnO, it also has negative effects on nutrient digestibility, effectiveness of phytase and organic acids, and is implicated in the occurrence of antibiotic resistance (Ciesinski et al., 2018). In this context, identifying alternative nutritional strategies for managing newly weaned pigs is an important issue across the globe. Utility of postbiotic enzymatically treated non-GMO *Saccharomyces cerevisiae* is among the dietary strategies that has been proposed to be beneficial in transitioning piglets upon weaning (Kiarie et al., 2011). Yeast β-glucans and mannan oligosaccharides are functional and are responsible for the immunomodulating properties and prevention of pathogenetic bacteria (e.g., *Escherichia coli*) binding and proliferating on intestinal surfaces (Anwar et al., 2017). The mode of action differs from ZnO which improves growth performance by increasing feed intake and, therefore, alleviating the negative effects typically associated with the post-weaning growth lag (i.e., high intestinal pH, thinner mucosal layer, reduced expression of tight junction proteins; Pluske, 2016).

There is little available information on comparative efficacy of postbiotic yeast and pharmacological ZnO in nursery pig feed program. Moreover, growth promoting mechanisms of the two ingredients may differ considerably. The objective of this study was to evaluate the efficacy of enzymatically treated yeast (HY40) as an alternative to pharmacological ZnO and if there were any additive effects of providing both HY40 and ZnO to nursery pigs post-weaning.

## Materials and Methods

Animal care and use protocols were approved by the University of Guelph Animal Care and Use Committee and pigs were cared for in accordance with the Canadian Council on Animal Care guidelines (CCAC, 2009).

### Animals and housing

A total of 144 (Yorkshire*Landrace ♀ X Duroc ♂) 21-d-old, weaned pigs (72 barrows and 72 gilts, 6.93 ± 0.48 kg initial body weight; BW) were procured from the University of Guelph Arkell Swine Research Station (Guelph, ON, Canada). Based on weaning BW, piglets were randomly assigned to pens (*n* = 4 piglets/pen, 2 barrows and 2 gilts) in two environmentally controlled rooms at Arkell swine research station. Each room had 18 pens equipped with a feeder, a nipple type drinker, plastic-covered expanded metal floors, and a wall partitioning between pens that allowed visual contact with pigs in adjacent pens. Room temperature was initially set at 29.5 °C and reduced by 1.5 °C per week for the first 4 wk after weaning.

### Diets and experimental procedures

At weaning, pigs were randomly assigned to one of four dietary treatment groups in a factorial arrangement: 1) control corn-wheat-soybean meal diet (Control); 2) control + HY40 (HY40); 3) control + zinc oxide (ZnO), and 4) control + HY40 + zinc oxide (HY40 + ZnO). During phase I, HY40 and/or ZnO was included in the diet at either 0.5% or 3,000ppm, respectively, and was halved in phase II. The HY40 was an enzymatically treated, whole, non-GMO *Saccharomyces cerevisiae* strain assayed to contain 40% β-1.3/1.6-glucans and mannan oligosaccharides (cell wall components) and 36% crude protein (Livalta TMCell HY40, AB Agri Ltd., Peterborough, UK). Diets were formulated to meet specifications of NRC (2012) in two phases: phase I (weeks 1 and 2) and phase II (weeks 3 to 6; NRC, 2021). All diets included 0.2% TiO_2_ as an indigestible marker. The diets were allocated to pens in a completely randomized block (room) design to give 9 replicates per diet. Pigs had free access to feed and water for 6 wk. Feed intake and BW were monitored weekly for calculation of ADG, ADFI, and G: F. The occurrence and severity of diarrhea were monitored and assessed on a pen basis using a fecal consistency scoring system (1-liquid, 2-soft, 3-normal, 4-hard) daily for the first 7 d post-weaning (days 0 to 7; Kiarie et al., 2011). Fresh fecal samples were taken on days 7, 14, and 28 for determination of fecal dry matter (DM) and apparent total tract digestibility (ATTD) of nutrients. At the end of phase I (day 14), one pig per pen was randomly selected, bled, and killed for organ weight and sampled for digesta and tissues. Blood samples (10 mL) were collected via the orbital sinus bleeding technique (Dove and Alworth, 2015) using a Monoject Standard Hypodermic needle 16 G × 1″ (Covidien; Mansfield, MA) into vacutainer tubes coated with lithium heparin (Becton Dickinson & Co, Franklin Lakes, NJ). The samples were immediately centrifuged at 2,000 × *g* for 10 min at 4 °C to recover plasma, which was immediately stored at −80 °C until used for analyses. The pig was subsequently sedated with a premix of 0.2 mL/kg BW [1 mL contained: Ketamine (50 mg), butorphanol (1 mg), and xylazine (10 mg)] via intramuscular injection followed by intravenous injection of Pentobarbital (Euthansol) at 68 mg/kg BW. The spleen and liver were removed, blotted dry with paper towels, weighed, and discarded. The mid-jejunum was located (approximately 1.5 m distal to the ligament of Trietz) and two segments were cut. One 3-cm jejunal segment was put in 10% formalin for histomorphology analysis and the other segment (1 cm) was rinsed with sterile saline (0.9% NaCl) and placed in a 2-mL tube filled with 1.2 mL Ambion RNA later (Life Technologies Inc., Burlington, ON, Canada). The samples were then placed on ice and transported to the lab and stored at −80 °C until required for mRNA analyses of digestive enzymes, nutrients transporters, tight junction proteins, and cytokines. Terminal ileum sections (5 cm anterior to ileal-ceca junction) were collected using BioFreeze sampling kits following the recommended protocol by the manufacturer and shipped to Alimetrics laboratories Ltd (Espoo, Finland) for further processing and analysis.

### Laboratory analyses

#### Nutrient digestibility

Fresh fecal samples were oven dried at 60 °C for 72 h. The weight before and after drying was recorded to calculate fecal dry matter (DM; Kiarie et al., 2009). Diet and fecal samples were finely ground (CBG5 Smart Grind, Applica Consumer Products Inc., Shelton, CT) and thoroughly mixed for analyses of DM, crude protein (CP), gross energy (GE), titanium, and Zn. Dry matter was determined using method 930.15 (AOAC, 2005) and nitrogen (N) analysis via combustion method 968.06 using a CNS-2000 carbon, N, and sulfur analyzer (LECO-FP 828 analyzer, LECO Instruments Ltd, Mississauga, ON, Canada). Crude protein values were derived by multiplying the assayed N values by a factor of 6.25. Gross energy was determined using a bomb calorimeter (IKA Calorimeter System C 5000; IKA Works, Wilmington, NC). Crude fat content was determined using an ANKOM XT 20 Extractor (Ankom Technology, Fairport, NY). Titanium concentration was measured using the method of Myers et al. (2004). Zn concentration was determined using inductively coupled plasma mass spectrophotometry (AOAC, 2005; method 985.01).

#### Microbial activity

The analysis of dominant bacterial species as well as short-chain fatty acids (SCFA) profiles was performed by Alimetrics Diagnostics Ltd (Espoo, Finland). Ileal digesta microbial concentration was conducted using quantitative real-time polymerase chain reaction (qPCR) method (Agyekum et al., 2016). Briefly, the samples were washed to remove solid particles and complex polysaccharides to improve subsequent DNA purification and the downstream qPCR applications. The liquid phase was subjected to differential centrifugation for collecting the bacterial cells. The microbial cell walls were disrupted, and the chromosomal DNA was quantitatively extracted and quantified using a Nanodrop 2000 spectrophotometer (ThermoScientific, Wilmington, DE). The qPCR of microbial analyses was conducted with 16S rRNA gene-targeted DistaMap analysis panel using SYBR Green chemistry method (Tajadini et al., 2014). The method is based on the detection and quantification of a fluorescent reporter signal that increases in direct proportion to the amount of polymerase chain reaction (PCR) product in the reaction. By recording the amount of fluorescence emission at each cycle, it is possible to monitor the PCR reaction during exponential phase where the first significant increase in the amount of PCR product correlates to the initial amount of target template. The present analyses targeted abundance of total bacteria, total *Lactobacilli (LAB)*, *Lactobacilli amylovorus*, *Lactobacilli reuteri*, *Lactobacilli johnsonii*, Streptococcus, Enterococcus, and *E. coli*. The primers for the target microbiota assessed in present study were previously reported (Kettunen et al., 2017; Apajalahti et al., 2019). The data were reported as number of copies of 16S rRNA per gram of sample. Short-chain fatty acids (lactic, acetic, propionic, iso-butyric, butyric, 2-methylbutyric, isovaleric, and valeric) were derivatized to the respective phenyl esters by using phenyl chloroformate reagent and analyzed by gas chromatography (Agilent Technologies, Santa Clara, CA) using pivalic acid (Sigma–Aldrich, St. Louis, MO) as an internal standard. The chromatography procedure used a glass column packed with 80/120 Carbopack B-DA/4% Carbowax stationary phase, helium as a carrier gas, and a flame ionization detector has been described previously by Apajalahti et al. (2019).

#### Jejunal gene expression

Total RNA from 50-100 mg of jejunal tissues was extracted using the Trizol method (Thermo Fisher Scientific, Mississauga, ON, Canada) following the manufacturer’s instruction. The RNA was purified by precipitation with lithium chloride and quantified by Nanodrop ND-2000 spectrophotometer (Thermo Fisher Scientific). The ratio of OD260 and OD280 was between 1.8 and 2.1. The integrity of RNA was verified by visualization in an agarose gel. The RNA samples were stored under −80 °C. To create a cDNA library, 2 μg of total RNA was reverse transcribed into cDNA using the Superscript II kit (Bio-Rad Hercules, CA) following the manufacturer’s instruction. The primers for real-time polymerase chain reaction (RT-PCR) analysis were designed with Primer-Blast based on the published cDNA sequence in the DNA bank or synthesized based on the primer sequences from publications (Table 1). All the primers spanned at least two exons. The primers were synthesized by Integrated DNA Technologies, Inc. The RT-PCR was performed using SYBR Green Supermix (Bio-Rad) on a CFX Connect Real-Time PCR Detection System (Bio-Rad). Two microliters of cDNA was added to a total volume of 20 μL containing 10 μL SYBR Green mix, and 1 μL each of forward and reverse primers. Each sample was analyzed in duplicate for each gene. The following thermocycling amplification conditions were used: denaturation 15 s at 95 °C, annealing 15 s at 56 °C, extension 30 s at 72 °C, repeating for 40 cycles. A melting curve program was conducted to confirm the specificity of each product. Real-time-PCR data were analyzed using the 2^−ΔΔCT^ method to calculate the relative fold change of target gene with GAPDH as internal control (Livak and Schmittgen, 2001).

**Table 1.** Gene-specific primers used for the analysis of mRNA levels using quantitative real-time RT-PCR in the ileum 14 d post-weaning

Gene	Genbank accession number	Product size (bp)	Primer sequence (5ʹ-3ʹ)	
*β-actin*	DQ845171	100	Forward	CACGCCATCCTGCGTCTGGA
			Reverse	AGCACCGTGTTGGCGTAGAG
*IL-6*	M86722	151	Forward	AAGGTGATGCCACCTCAGAC
			Reverse	TCTGCCAGTACCTCCTTGCT
*TNFα*	X54001	151	Forward	ATGGATGGGTGGATGAGAAA
			Reverse	TGGAAACTGTTGGGGAGAAG
*EAAC-1*	NM_001164649.1	168	Forward	CCTCAGTGGTGCTAGGGATTG
			Reverse	GGGCAGCAACACCTGTAATC
*B* ^*0*^ *AT-1*	XM_003359855	102	Forward	AAGGCCCAGTACATGCTCAC
			Reverse	CATAAATGCCCCTCCACCGT
*SGLT-1*	XM_021072101.1	153	Forward	GGCTGGACGAAGTATGGTGT
			Reverse	ACAACCACCCAAATCAGAGC
*PepT-1*	NM_214347	143	Forward	CATCGCCATACCCTTCTG
			Reverse	TTCCCATCCATCGTGACATT
*ZO-1*	XM_021098856	200	Forward	GATCCTGACCCGGTGTCTGA
			Reverse	TTGGTGGGTTTGGTGGGTTG
*OCLN*	NM_001163647	163	Forward	GAGAGAGTGGACAGCCCCAT
			Reverse	TGCTGCTGTAATGAGGCTGC
*IL-10*	NM_214041	220	Forward	CATCCACTTCCCAACCAGCC
			Reverse	CTCCCCATCACTCTCTGCCTTC
*SOD-1*	NM_001190422	104	Forward	GTACCAGTGCAGGTCCTCAC
			Reverse	TTTGCCAGCAGTCACATTGC

B^0^AT1, sodium-dependent neutral amino acid transporter; EAAC1, excitatory amino-acid carrier 1; PepT1, human peptide transporter 1; SGLT1, sodium-glucose cotransporter 1; ZO-1, Zonula occuldens-1; OCLN, Occludin; SOD-1, superoxide dismutase.

#### Histomorphology

Jejunal segments were prepared for analysis according to Carleton et al. (1980). Approximately 3–4 cross sections of the jejunum were used to measure villus height (VH), villus width (VW), crypt depth (CD) and crypt width (CW) per pig. On each cross section, 10 replicates per parameter were recorded and average values for VH and CD were used to calculate VH:CD. Average values for VH, VW, and CW were used to calculate absorptive capacity (M; equation 1) according to (Kisielinski et al., 2002),


$$\begin{array}{l} M=\frac{\left(VW\times \;VH\right)+{\left(\frac{VW}{2}+\frac{CW}{2}\right)}^{2}-{\left(\frac{VW}{2}\right)}^{2}}{{\left(\frac{VW}{2}+\frac{CW}{2}\right)}^{2}} \end{array}$$ (1)

#### IgA assay in jejunal tissues and plasma

Frozen jejunal samples were ground using mortar and pestle in liquid nitrogen. An aliquot of pulverized jejunal tissue samples (0.12 ± 0.022 g) were placed into a free-standing microcentrifuge tube (02-682-558, Thermo Fisher, Waltham, MA) followed by addition of Tissue Protein Extraction Reagent (T-PER; sample weight × 15; 78510, Thermo Fisher, Waltham, MA; Kakhki et al., 2020). Then, 0.1 ± 0.01 g of acid-washed glass beads (≤ 106 µm; G4649-100G, Sigma Aldrich, St. Louis, MO) were added and followed by homogenization with a bead mill for two cycles of 150 s at 3 m/s (15-340-163; Fisher Brand bead mill-24, Thermo Fisher, Waltham MA). Homogenized samples were then centrifuged at 10,000 × *g* for 15 min at 4 °C (Kakhki et al., 2020). Supernatants of jejunal tissue homogenates and thawed plasma were used for assay for the concentration of IgA using commercial pig IgA ELISA kits according to the manufacturer instructions (Cedarlane Labs., Burlington, ON, Canada).

#### Calculations and statistical analyses

Apparent total tract digestibility (ATTD) of energy and nutrients were calculated using marker standard method (Adeola, 2000). The microbial data were log transformed before statistical analyses. Data were evaluated for the presence of outliers using box and whisker method and subsequently subjected to Mixed model of the GLIMMIX procedure of SAS (University Edition; SAS Inst. Inc., Cary, NC), with pen as the experimental unit. The model had main effects of HY40 and ZnO and associated interactions as fixed effects and block (room) as the random effect. Independent t-test and Tukey methods were used to separate LSmeans for the main and interaction effects, respectively. In all analyses, degrees of freedom were calculated with Satterthwaite for general linear mixed model. An α level of *P* ≤ 0.05 was considered significant.

## Results

Analyzed chemical composition of the experimental diets (Table 2) was largely consistent with formulated values.

**Table 2.** Ingredient composition and calculated and analyzed nutrient contents of nursery diets (as-fed basis)^1^

Item	Phase I				Phase II			
	Control	HY40^1^	ZnO^1^	HY40 +ZnO^1^	Control	HY40	ZnO	HY40 +ZnO
Ingredient, %								
Corn	39.9	39.4	39.5	39.0	42.2	41.9	42.0	41.7
Wheat	10.0	10.0	10.0	10.0	15.0	15.0	15.0	15.0
Soybean meal 46%	29.9	29.9	29.9	29.9	22.1	22.1	22.1	22.1
Soy oil	2.40	2.40	2.40	2.40	1.57	1.57	1.57	1.57
Canola meal	2.50	2.50	2.50	2.50	5.00	5.00	5.00	5.00
Barley	5.00	5.00	5.00	5.00	10.0	10.0	10.0	10.0
L-Lysine HCL	0.46	0.46	0.46	0.46	0.50	0.50	0.50	0.50
DL-Methionine	0.17	0.17	0.17	0.17	0.12	0.12	0.12	0.12
L-Threonine	0.16	0.16	0.16	0.16	0.15	0.15	0.15	0.15
L-Valine	0.05	0.05	0.05	0.05	0.04	0.04	0.04	0.04
Whey permeate	5.95	5.95	5.95	5.95	–	–	–	–
Limestone	1.08	1.08	1.08	1.08	0.96	0.96	0.96	0.96
Monocalcium phosphate	0.90	0.90	0.90	0.90	0.75	0.75	0.75	0.75
Salt	0.36	0.36	0.36	0.36	0.44	0.44	0.44	0.44
Titanium dioxide (TiO_2_)	0.20	0.20	0.20	0.20	0.20	0.20	0.20	0.20
Vitamins and trace minerals premix^2^	1.00	1.00	1.00	1.00	1.00	1.00	1.00	1.00
Phytase^3^	0.01	0.01	0.01	0.01	0.01	0.01	0.01	0.01
Zinc oxide, 72%	–	–	0.39	0.39	–	–	0.20	0.20
HY40^4^	–	0.50		0.50	–	0.25	–	0.25
Calculated provisions^4^								
NE, kcal/kg	2,448	2,448	2,448	2,448	2,412	2,412	2,412	2,412
Crude protein, %	20.6	20.6	20.6	20.6	18.9	18.9	18.9	18.9
SID Lys, %^5^	1.35	1.35	1.35	1.35	1.23	1.23	1.23	1.23
Total Phosphorus, %	0.62	0.62	0.62	0.62	0.57	0.57	0.57	0.57
Std. Dig. P, %	0.40	0.40	0.40	0.40	0.35	0.35	0.35	0.35
Ca, %	0.80	0.80	0.80	0.80	0.70	0.70	0.70	0.70
Phytase, FTU/kg	500	500	500	500	500	500	500	500
Zinc, ppm	227	227	3,000	3,000	232	232	1,500	1,500
Analyzed composition, %								
Gross energy, kcal/kg	4,083	4,109	4,085	4,064	4,077	4,022	3,998	4,008
Crude protein, %	20.5	22.8	20.8	22.1	18.7	19.7	18.6	19.2
Calcium, %	0.80	0.82	0.81	0.71	0.76	0.76	0.62	0.71
Phosphorous, %	0.60	0.61	0.56	0.56	0.53	0.52	0.52	0.58
Zinc, ppm	242	215	2,969	2,902	325	264	1,464	1,563

Dietary treatments: Control; HY40; ZNO; HY40+ZNO.HY40 included in HY40 and HY40+ZnO diets at 0.5% in phase I (0–14 d post-weaning) and 0.25% in phase II (15–42 d post-weaning); ZnO included in ZnO and HY40+ZnO diets at 3,000 ppm in phase I (0–14 d post-weaning) and 1,500 ppm in phase II (15–42 d post-weaning).

Provided per kg of premix: vitamin A, 2,000,000 IU as retinyl acetate; vitamin D_3_, 200,000 IU as cholecalciferol; vitamin E, 8,000 IU as dl-α-tocopherol acetate; vitamin K, 500 mg as menadione; pantothenic acid, 3,000 mg; riboflavin, 1,000 mg; choline, 100,000 mg; folic acid, 400 mg; niacin, 5,000 mg; thiamine, 300 mg; pyridoxine, 300 mg; vitamin B_12_, 5,000 mcg; biotin, 40,000 mcg; Cu, 3,000 mg from CuSO_4_×5H_2_O; Fe, 20,000 mg from FeSO_4_; Mn, 4,000 mg from MnSO_4_; Zn, 21,000 mg from ZnO; Se, 60 mg from Na_2_SeO_3_; and I, 100 mg from KI (DSM Nutritional Products Canada Inc., Ayr, ON, Canada).

Provided 0.15% available P and 0.16% Ca (Quantum Blue, AB Vista, Marlborough, UK).

Enzymatically treated whole non-GMO Saccharomyces cerevisiae strain assayed to contain 40% β-1.3/1.6 glucans and mannan oligosaccharides and 36% crude protein (Livalta TMCell HY40, AB AGRI, Peterborough, UK).

Standarized ileal digestible.

### Growth performance

There was no (*P* > 0.05) interaction between HY40 and ZnO on BW and ADG. Feeding HY40 had no impact on ADG in phase I (300 vs. 285 g/d; *P* = 0.178); however, pigs fed diets with HY40 exhibited higher ADG than without HY40 in phase II (759 vs. 734 g/d; *P* = 0.031) and overall (530 vs. 509 g/d; *P* = 0.018; Table 3). As such, the effects of HY40 on BW were detected in phase II, specifically, in weeks 5 (*P* = 0.020)  and 6 (*P* = 0.025) with piglets fed diets with HY40 being heavier than pigs fed diets without HY40. Consequently, pigs fed with HY40 were 1 kg (32.8 vs. 31. 8 kg; *P =* 0.025) heavier than pigs fed without HY40 at the end of the trial.

**Table 3.** Comparative evaluation of treated yeast (HY40) and/or pharmacological zinc oxide (ZnO) on growth performance, and organ weights in nursery pigs from weaning to 42 d post-weaning^1^

	Main effects					Interaction effects					*P*-value^2^		
	Without HY40	With HY40	Without ZnO	With ZnO	SEM^3^	Control	HY40	ZnO	HY40+Zinc	SEM^2^	HY40	ZnO	HY40×ZnO
No.^4^	18	18	18	18		9	9	9	9				
Body weight, kg													
Day 0	7.3	7.3	7.4	7.2	0.07	7.4	7.3	7.2	7.3	0.11	0.734	0.232	0.417
Day 7	8.1	8.3	8.1	8.3	0.09	8.1	8.1	8.2	8.5	0.13	0.230	0.124	0.215
Day 14	11.3	11.5	10.9	11.9	0.14	10.9	10.9	11.7	12.2	0.20	0.216	<0.01	0.323
Day 21	15.2	15.5	14.6	16.1	0.19	14.7	14.5	15.6	16.5	0.26	0.273	<0.01	0.056
Day 28	20.0	20.4	19.4	21.0	0.24	19.5	19.4	20.5	21.4	0.33	0.269	<0.01	0.114
Day 35	25.9	26.8	25.4	27.4	0.24	25.3	25.5	26.6	28.1	0.34	0.020	<0.01	0.073
Day 42	31.8	32.8	31.3	33.3	0.30	31.1	31.4	32.4	34.1	0.42	0.025	<0.01	0.112
ADG, g/d													
Phase I	285	300	250	335	7.75	247	254	324	347	10.9	0.178	<0.01	0.479
Phase II	734	759	732	760	8.05	728	737	739	782	11.4	0.031	0.021	0.142
Overall	509	530	491	548	5.78	487	495	531	564	8.16	0.018	<0.01	0.136
ADFI, g/d													
Phase I	359	372	327	404	14.0	316	338	402	405	19.8	0.537	<0.01	0.637
Phase II	1215	1265	1199	1281	17.4	1213^b^	1186^b^	1217^b^	1345^a^	24.6	0.050	0.002	0.004
Overall	787	818	763	842	13.4	764	762	810	875	18.9	0.107	<0.01	0.084
G:F													
Phase I	0.80	0.81	0.77	0.84	0.02	0.78	0.76	0.82	0.86	0.03	0.863	0.030	0.334
Phase II	0.60	0.60	0.61	0.60	0.01	0.60	0.62	0.61	0.58	0.01	0.897	0.157	0.070
Overall	0.70	0.71	0.69	0.72	0.01	0.69	0.69	0.72	0.72	0.02	0.915	0.145	0.823
Organ weight, g/kg of BW													
Small intestine	54.3	52.8	55.0	52.1	1.76	55.1	54.9	53.5	50.7	2.48	0.551	0.259	0.609
Liver	31.5	33.6	32.9	32.3	1.18	32.7	33.0	30.4	34.1	1.66	0.227	0.712	0.310
Spleen	2.60	2.75	2.59	2.75	0.17	2.38	2.81	2.81	2.69	0.23	0.518	0.509	0.256

Dietary treatments: Control; HY40; ZNO; HY40+ZNO.HY40 included in HY40 and HY40+ZnO diets at 0.5% in phase I (0–14 d post-weaning) and 0.25% in phase II (15–42 d post-weaning); ZnO included in ZnO and HY40+ZnO diets at 3,000 ppm in phase I (0–14 d post-weaning) and 1,500 ppm in phase II (15–42 d post-weaning).

*P*-values for the main and interactive effects between ZnO and HY40.

Maximum value for the standard error of the means.

Number of pens evaluated.

Means without a common superscript differ, *P* < 0.05.

Pigs fed diets with ZnO had higher (*P* < 0.01) ADG than pigs fed diets without ZnO throughout the trial and were consequently heavier (*P* < 0.01) than pigs fed non-ZnO diets from weeks 2 to 6. There was an interaction (*P* = 0.004) between HY40 and ZnO on ADFI in phase II, such that pigs fed HY40+ZnO combination showed greater ADFI than those fed control or HY40 or ZnO. ADFI of pigs fed with HY40 followed the pattern of growth response, exhibiting no effect (*P* = 0.537) in phase I but significant effect (1265 vs. 1215 g/d; *P* = 0.050) in phase II compared to pigs fed diets without HY40. Pigs fed diets with ZnO showed greater  (*P* < 0.01) ADFI throughout the trial corresponding to higher growth rates. Pigs fed diets with ZnO were heavier by day 14 and remained heavier than pigs fed diets without ZnO until the end of the study (*P <* 0.01). Additionally, ZnO improved phase I G:F (*P* = 0.030) but not G:F during phase II or overall.

#### Nutrient digestibility and gut physiology

Further evaluation of organ weights (small intestine, liver, and spleen) revealed no effects (*P* > 0.05) of diet (Table 3). We observed an interaction between HY40 and ZnO on fecal dry matter such that pigs fed HY40 had drier feces  (*P =* 0.01) on day 14 suggesting reduced incidence of diarrhea than from the combination of HY40 + ZnO, ZnO, or control (Table 4). On day 14, control-fed pigs had lower (*P* < 0.05)  ATTD of DM and GE than pigs fed HY40 and ZnO alone or combined. However, on day 28, pigs fed ZnO had lower (*P* < 0.05) ATTD of DM, and GE than all other dietary treatments, and ATTD of CP was lower for pigs given ZnO than HY40 or HY40 + ZnO, with those provided the control diet intermediate.

**Table 4.** Comparative evaluation of treated yeast (HY40) and/or pharmacological zinc oxide (ZnO) on fecal dry matter content (g/kg) and apparent total tract digestibility (ATTD, %) of nutrients and energy in nursery pigs^1^

	Main effects					Interaction effects					*P*-value^2^		
	Without HY40	With HY40	Without ZnO	With ZnO	SEM^3^	Control	HY40	ZnO	HY40+ ZnO	SEM^2^	HY40	ZnO	HY40×ZnO
No.^4^	18	18	18	18		9	9	9	9				
Fecal consistency scores^4^													
Day 0	2.7	2.7	2.7	2.6	0.07	2.7	2.7	2.6	2.6	0.10	0.778	0.261	0.940
Day 1	3.1	3.0	2.9	3.1	0.08	3.1	2.8	3.1	3.1	0.11	0.257	0.109	0.271
Day 2	2.8	2.8	2.7	2.9	0.07	2.7	2.7	2.97	2.9	0.10	0.794	0.090	0.577
Day 3	2.8	2.8	2.8	2.9	0.07	2.7	2.8	2.9	2.8	0.10	0.995	0.383	0.383
Day 4	2.7	2.7	2.6	2.7	0.09	2.6	2.7	2.7	2.7	0.13	0.934	0.598	0.598
Day 5	2.6	2.5	2.5	2.6	0.10	2.6	2.5	2.6	2.6	0.14	0.519	0.734	0.911
Day 6	2.5	2.5	2.4	2.6	2.51	2.4	2.4	2.6	2.6	0.15	0.909	0.386	0.979
Day 7	2.6	2.6	2.4	2.8	0.08	2.4	2.5	2.8	2.8	0.12	0.969	0.007	0.798
Overall	2.7	2.7	2.6	2.8	0.06	2.7	2.6	2.8	2.7	0.08	0.719	0.150	0.850
Fecal dry matter, g/kg													
Day 7	271	286	268	289	11.46	267	270	275	302	15.3	0.370	0.228	0.460
Day 14	266	296	310	253	16.30	265^b^	355^a^	268^b^	237^b^	21.8	0.210	0.020	0.014
Day 28	303	289	290	302	6.84	302	279	305	298	9.16	0.140	0.248	0.408
Fecal Zn, mg/kg DMI													
Day 7	1213	1403	282	2334	172.9	330	245	2097	2571	209.0	0.285	<0.001	0.115
Day 14	803	1182	311	1675	141.3	162	460	1445	1904	154.2	0.013	<0.001	0.573
Day 28	1109	1008	278	1839	126.4	298	259	1920	1757	132.2	0.432	<0.001	0.627
ATTD, %													
Day 7													
Dry matter	78.4	78.4	77.9	78.9	1.08	77.4	78.3	79.4	78.5	1.52	0.995	0.487	0.537
Crude protein	66.5	66.7	65.5	67.7	2.01	65.1	66.0	68.0	67.4	2.84	0.953	0.459	0.792
Gross energy	75.4	75.9	74.6	76.7	1.28	73.7	75.5	77.1	76.3	1.81	0.777	0.262	0.463
Day 14													
Dry matter	82.8	83.9	82.5	84.2	0.30	81.3^b^	83.7^a^	84.3^a^	84.1^a^	0.42	0.016	0.0001	0.003
Crude protein	75.2	77.4	73.9	78.8	0.56	72.2	75.5	78.2	79.4	0.8	0.009	<0.001	0.195
Gross energy	81.5	83.3	81.1	83.8	0.33	79.5^b^	82.6^a^	83.6^a^	84.0^a^	0.8	0.001	<0.001	0.009
Day 28													
Dry matter	80.1	81.9	81.8	80.2	0.54	81.9^a^	81.6^a^	78.2^b^	82.2^a^	0.77	0.022	0.051	0.009
Crude protein	69.4	73.5	73.2	69.7	1.09	72.3^ab^	74.2^a^	66.6^b^	72.9^a^	1.54	0.012	0.032	0.016
Gross energy	79.8	81.3	81.5	79.6	0.57	81.7^a^	81.2^a^	77.9^b^	81.3^a^	0.81	0.082	0.029	0.022

Dietary treatments: Control; HY40; ZNO; HY40+ZNO.HY40 included in HY40 and HY40+ZnO diets at 0.5% in phase I (0–14 d post-weaning) and 0.25% in phase II (15–42 d post-weaning); ZnO included in ZnO and HY40+ZnO diets at 3,000 ppm in phase I (0–14 d post-weaning) and 1,500 ppm in phase II (15–42 d post-weaning).

*P*-values for the main and interactive effects between ZnO and HY40.

Maximum value for the standard error of the means.

Number of pens evaluated.

Fecal consistency scores: 1-liquid, 2-soft, 3-normal, 4-hard.

Means without a common superscript differ, *P* < 0.05.

Fecal consistency scores were not different between dietary treatments during the first 7 d post-weaning, apart from day 7 in which pigs fed diets with ZnO had a higher fecal consistency score, indicating they had firmer feces than pigs given diets without ZnO (Table 4). Fecal dry matter on days 7 and 28 post-weaning was not different between treatment groups. On day 14, there was an interaction between HY40 and ZnO such that pigs fed HY40 had greater fecal dry matter than ZnO, HY40 + ZnO, or the control.

There were no interactive effects between HY40 and ZnO on fecal Zn excretion *(P* = 0.1146, *P* = 0.5733, *P* = 0.6267) for days 7, 14, and 28, respectively (Table 4). On days 7, 14, and 28, the main effect of ZnO resulted in greater Zn excretion in pigs fed diets with ZnO versus without ZnO  (*P* < 0.001). On day 14, the main effect of HY40 also impacted fecal Zn excretion such that pigs fed diets with HY40 had greater fecal Zn excretion than pigs fed diets without HY40 (*P* = 0.0128). Although the interaction between HY40 and ZnO was not significant on day 14, the significant main effect of HY40 on Zn excretion seems to be due to the inclusion of HY40 + ZnO in the HY40-containing diets. Pigs fed HY40 alone had a low fecal Zn excretion (456 mg/kg dry matter intake; DMI) and when averaged between HY40 and HY40 + ZnO (1,904 mg/kg DMI) this resulted in a significant difference attributed to ZnO on day 14.

### Ileal digesta concentrations of short-chain fatty acids and microbiota

An interaction between HY40 and ZnO on ileal digesta concentration of *E. coli* indicated that pigs fed HY40 + ZnO showed lower (*P* < 0.05) *E. coli* gene abundance than piglets fed control or HY40, with pigs fed ZnO intermediate between these (Table 5). There was no effect (*P* > 0.05) on ileal digesta total or individual SCFA attributed to HY40 or the interaction between HY40 and ZnO. Pigs fed diets with ZnO had lower total SCFA (26.2 vs. 46.0 mmol/kg; *P* = 0.04) but greater concentrations of VFA (7.33 vs. 4.67 mmol/kg;  *P* = 0.010). Pigs fed diets with ZnO showed higher (*P* = 0.004) concentration of *Enterococcus* (*P* = 0.001) and lower *E. coli* in ileal digesta (*P* = 0.0002). This resulted in greater LAB: *E. coli* ratio (1.44 vs. 1.20; *P* = 0.001) for pigs fed with ZnO versus diets without ZnO. Pigs fed ZnO-containing diets had higher acetic (6.61 vs. 4.45 mmol/kg; *P* = 0.01) and butyric acid (0.30 vs. 0.09 mmol/kg; *P* = 0.01) but lower lactic acid (18.9 vs. 41.3 mmol/kg; *P*=0.02) concentrations than piglets fed without ZnO. No differences in SCFAs were attributed to feeding HY40-containing diets.

**Table 5.** Comparative evaluation of treated yeast (HY40) and/or pharmacological zinc oxide (ZnO) on ileal digesta concentration of microbiota and mid-colon concentration of short-chain fatty acids (SCFA) in nursery pigs 14 d post-weaning^1^

	Main effects					Interaction effects					*P*-value^2^		
	Without HY40	With HY40	Without ZnO	With ZnO	SEM^3^	CON	HY40	ZnO	HY40+ ZnO	SEM^2^	HY40	ZnO	HY40×ZnO
No.^4^	18	18	18	18		9	9	9	9				
Microbiota, log10 16S genes/g of digesta (wet)													
Total bacteria	11.04	11.08	10.92	11.20	0.19	10.89	10.94	11.20	11.21	0.23	0.855	0.123	0.917
Total *Lactobacilli* (LAB)	10.64	10.69	10.55	10.79	0.18	10.44	10.65	10.84	10.74	0.26	0.846	0.367	0.556
*Lactobacilli amylovorus*	9.48	9.67	9.68	9.47	0.27	9.35	10.01	9.60	9.33	0.38	0.628	0.582	0.234
*Lactobacilli reuteri*	9.54	9.91	9.68	9.76	0.24	9.57	9.79	9.50	10.02	0.24	0.139	0.723	0.543
*Lactobacilli johnsonii*	9.33	9.39	9.25	9.48	0.20	9.31	9.19	9.36	9.60	0.29	0.834	0.432	0.527
*Streptococcus*	9.21	9.17	9.05	9.33	0.29	9.16	8.93	9.26	9.41	0.35	0.875	0.306	0.496
*Enterococcus*	7.98	7.97	7.42	8.52	0.26	7.33	7.52	8.62	8.43	0.34	0.997	0.001	0.559
*E. coli*	8.55	8.06	8.89	7.71	0.40	8.75^a^	9.03^a^	8.34^ab^	7.09^b^	0.47	0.178	0.002	0.039
LAB: *E. coli*	1.27	1.37	1.20	1.44	0.07	1.20	1.18	1.33	1.56	0.07	0.127	0.001	0.077
SCFA, mmol/kg													
Total SCFA^5^	31.22	41.00	45.99	26.23	6.25	37.06	54.93	25.39	27.07	8.84	0.277	0.033	0.367
Acetic acid	5.67	5.49	4.45	6.61	0.94	4.62	4.27	6.51	6.71	1.09	0.925	0.010	0.729
Propionic acid	0.28	0.14	0.16	0.26	0.14	0.12	0.21	0.44	0.07	0.19	0.441	0.609	0.212
Butyric acid	0.17	0.22	0.09	0.30	0.11	0.14	0.05	0.21	0.39	0.12	0.584	0.019	0.131
Valeric acid	0.04	0.03	0.03	0.04	0.01	0.03	0.03	0.05	0.03	0.02	0.519	0.377	0.605
Lactic acid	25.13	35.09	41.33	18.90	6.25	32.22	50.44	18.05	19.75	8.84	0.268	0.016	0.357
Total BCFA^6^	0.03	0.03	0.03	0.03	0.01	0.03	0.02	0.03	0.03	0.01	0.471	0.297	0.948
Total VFA^7^	6.09	5.91	4.76	7.23	1.17	4.94	4.59	7.24	7.22	1.33	0.842	0.010	0.857
SCFA: BCFA	1163	1629	1818	973	243.1	1461	2174	864	1083	343.8	0.185	0.020	0.478
Molar proportion^8^, %													
Acetic acid	27.00	26.56	18.50	35.06	6.06	24.38	12.62	29.62	40.50	7.36	0.941	0.009	0.065
Propionic acid	1.10	0.55	0.44	1.21	0.52	0.39	0.48	1.81	0.62	0.71	0.425	0.268	0.356
Butyric acid	0.92	1.01	0.59	1.34	0.66	0.99	0.18	0.85	1.84	0.74	0.848	0.107	0.055
Valeric Acid	0.17	0.16	0.10	0.24	0.05	0.12	0.07	0.22	0.25	0.07	0.890	0.034	0.513
Lactic Acid	70.65	71.61	80.22	62.04	7.08	73.92	86.52	67.39	56.70	8.36	0.880	0.007	0.073

Dietary treatments: control, HY40; ZNO; HY40+ZNO.HY40 included in HY40 and HY40+ZnO diets at 0.5% in phase I (0–14 d post-weaning) and 0.25% in phase II (15–42 d post-weaning); ZnO included in ZnO and HY40+ZnO diets at 3,000 ppm in phase I (0–14 d post-weaning) and 1,500 ppm in phase II (15–42 d post-weaning).

*P*-values for the main and interactive effects between ZnO and HY40.

Maximum value for the standard error of the means.

Number of pens evaluated.

Summation of acetic, propionic, butyric, valeric, and lactic acids.

Summation of iso-valeric and Isobutryic.

Summation of acetic, propionic, butyric, and valeric acids.

Concentration of SCFA divided by total SCFA expressed as percentage.

Means without a common superscript differ, *P* < 0.05.

### Nutrient transporters, tight junction protein, oxidative stress, and jejunal histomorphology

No differences (*P* > 0.05) were observed for the expression of genes for nutrient transporters (B^0^AT1, EAAC1, PepT1, SGLT1), tight junction proteins (ZO-1, OCLN), and oxidative stress (SOD-1) attributed to feeding HY40-containing diets (Table 6). Pigs provided diets with ZnO had greater jejunal expression of EAAC1, PepT1, SGLT1, and OCLN than pigs fed without ZnO (*P* < 0.05). No differences (*P* > 0.05) in cytokine gene expression were observed attributed to dietary treatment.

**Table 6.** Comparative evaluation of treated yeast (HY40) and/or pharmacological zinc oxide (ZnO) on jejunal expression of selected genes and jejunal histomorphology in nursery pigs 14 d post-weaning^1^

	Main effects					Interaction effects					*P*-value^2^		
	Without HY40	With HY40	Without ZnO	With ZnO	SEM^3^	Control	HY40	ZnO	HY40+ZnO	SEM^2^	HY40	ZnO	HY40×ZnO
No.^4^	18	18	18	18		9	9	9	9				
Nutrient transporters													
B^0^AT1	8.22	8.89	7.90	9.21	0.49	7.76	8.03	8.68	9.75	0.69	0.341	0.067	0.568
EAAC1	7.96	7.99	6.75	9.20	0.53	6.93	6.57	9.00	9.40	0.74	0.976	0.003	0.616
PepT1	6.94	7.82	6.46	8.30	0.51	6.13	6.79	7.76	8.85	0.72	0.230	0.016	0.768
SGLT1	5.44	6.15	4.93	6.66	0.49	4.63	5.23	6.26	7.07	0.69	0.317	0.019	0.876
Cytokines													
Il-6	13.52	14.51	13.39	14.64	0.48	12.86	13.92	14.17	15.1	0.67	0.151	0.075	0.927
IL-10	12.80	13.57	12.78	13.59	0.48	12.44	13.13	13.17	14.01	0.68	0.270	0.249	0.912
TNF- α	12.27	13.06	12.32	13.01	0.49	12.15	12.49	12.39	13.63	0.69	0.262	0.327	0.519
Tight junction proteins													
ZO-1	8.31	9.04	8.07	9.28	0.51	7.77	8.38	8.85	9.7	0.72	0.318	0.106	0.872
OCLN	7.30	8.05	6.77	8.58	0.55	6.35	7.19	8.25	8.91	0.78	0.340	0.028	0.904
Oxidative stress													
SOD-1	4.35	5.07	3.98	5.44	0.57	3.58	4.38	5.12	5.75	0.81	0.383	0.084	0.912
Histomorphology^5^													
VH, µm	746	756	743	759	17	719	767	773	744	25	0.696	0.531	0.128
CD, µm	224	200	208	215	5	222	195	226	204	7	0.002	0.346	0.726
VH:CD	3.34	3.82	3.61	3.55	0.10	3.26	3.97	3.43	3.66	0.13	0.002	0.636	0.089
VW, µm	120	122	118	125	4	113	122	128	122	6	0.767	0.205	0.215
CW, µm	40	39	38	42	2	37	38	44	40	2	0.447	0.016	0.101
M, µm^2^	14.5	14.8	15.1	14.2	0.48	14.9	15.3	14.0	14.4	0.7	0.635	0.203	0.984

B^0^AT1, sodium-dependent neutral amino acid transporter; EAAC1, excitatory amino-acid carrier 1; PepT1, human peptide transporter 1; SGLT1, sodium-glucose cotransporter 1; ZO-1, Zonula occuldens-1; OCLN, Occludin; SOD-1, superoxide dismutase; VH, villus height; CD, crypt depth; VW, villus width; CW, crypt width; M, absorptive capacity.

Dietary treatments: Control; HY40; ZNO; HY40+ZNO.HY40 included in HY40 and HY40+ZnO diets at 0.5% in phase I (0–14 d post-weaning) and 0.25% in phase II (15–42 d post-weaning); ZnO included in ZnO and HY40+ZnO diets at 3,000 ppm in phase I (0–14 d post-weaning) and 1,500 ppm in phase II (15–42 d post-weaning).

*P*-values for the main and interactive effects between ZnO and HY40.

Maximum value for the standard error of the means.

Number of pens evaluated.

Minimum of 30 villi and crypts were used to determine the villus height (VH), villus width (VW), crypt depth (CD), and crypt width (CW) per replicate.

Means without a common superscript differ, *P* < 0.05.

There were no interactive effects between HY40 and ZnO for any histomorphology measurements (*P >* 0.05). Provision of diets with HY40 did not affect VH but did reduce CD (200 vs. 224 μm; *P* = 0.002) resulting in increased VH:CD (3.82 vs. 3.34; *P* = 0.002). The VW and CW were not impacted by feeding HY40-containing diets, corresponding to M (absorptive capacity) not being different *(P* = 0.635). Feeding ZnO-containing diets did not impact VH, CD, or VW but did result in wider crypts (42 vs. 38 μm; *P* = 0.016). However, this did not correspond to a reduced M (*P* > 0.05).

### Immune response

**Figure 1.**
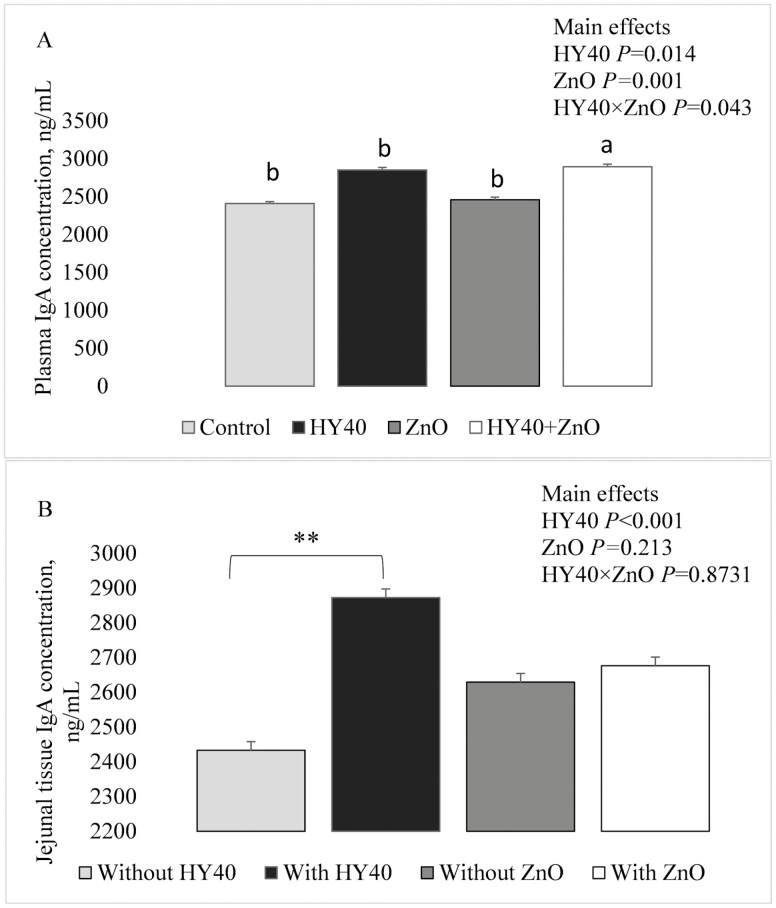
Plasma (A) and jejunal tissue (B) IgA concentrations (ng/mL) of pigs 14 d post-weaning fed diets with or without 0.5% enzymatically treated whole non-GMO *Saccharomyces cerevisiae* (HY40) or pharmacological levels (3,000 ppm) of zinc oxide (ZnO) or both (HY40 + ZnO). Values are Lsmeans ± SEM, *n* = 9. ^a,b^Means without a common superscript differ, *P* < 0.05. **Means with a *P* < 0.0001.

There were no differences in 16S rRNA expression of cytokines (IL-6, IL-10, TNF-α) in the jejunal tissues 14 d post-weaning (Table 6). An interactive effect of dietary treatment on plasma IgA concentrations such that pigs fed HY40 + ZnO had higher (*P =* 0.043) IgA concentration than any other treatment group 14 d post-weaning (Figure 1A). Additionally, the main effect of HY40 and ZnO also resulted in greater IgA plasma concentrations than pigs fed diets without HY40 or ZnO (71.2 vs. 54.5 ng/mL; *P* = 0.014 and 75.5 vs. 50.2 ng/mL; *P* = 0.001; respectively; data not shown). There were no interactive effects for jejunal tissue IgA concentrations and only pigs provided HY40-containing diets had higher jejunal IgA concentration than pigs fed diets without HY40  (*P* < 0.001; Figure 1B).

## Discussion

Following weaning, pigs typically experience a growth lag, defined as a period where post-weaning growth is less than pre-weaning growth. This is typically due to compounding stressors resulting from extremely low feed intake (Pluske et al., 2016). The objective of the current study was to determine the efficacy of HY40 compared to ZnO alone or in combination supplemented in nursery diets on growth performance, gut health (i.e., nutrient digestibility, histomorphology, SCFA, and oxidative stress) and immunoglobulin concentrations. Dietary supplementation of HY40 and ZnO improved nursery exit bodyweight (42 d post-weaning) over pigs fed diets without additives. Although no differences in growth performance were reported during phase I that was attributed to the inclusion of HY40, phase II ADG was improved when pigs were provided diets with HY40 or ZnO, and ADFI was also increased when HY40 + ZnO was provided. Additionally, feeding HY40 and ZnO alone, or in combination improved ATTD of DM and GE 14 d post-weaning compared to the control. The ATTD of CP on day 14 was also improved when pigs were fed diets with HY40 and with ZnO, and ATTD of DM and GE was greater for pigs fed ZnO alone than all other dietary treatments on day 28. Indices of gastrointestinal health (i.e., SCFA, oxidative stress, and tight junction proteins) were not affected by HY40 14 d post-weaning, but jejunal VH:CD and IgA were improved for pigs fed diets with HY40. Therefore, HY40 is effective in improving growth performance and gastrointestinal physiology (i.e., histomorphology and nutrient digestibility) in nursery pigs and could be used as an alternative for pharmacological feeding of ZnO.

The inclusion of yeast products in nursery diets has been previously studied; however, there are three different types of yeast additives which differ in their composition and efficacy. The three forms include live yeast (probiotic), separated yeast cell content and wall components (prebiotic), or inactived/ enzymatically treated whole yeast (postbiotic; Salminen et al., 2021). The term postbiotic is relatively new, but refers to non-living microorganisms, including whole yeast, also containing components such as cell walls and metabolites (Salminen et al., 2021). In the current study, a postbiotic yeast was tested, which supplied enzymatically treated β-glucans, mannan oligosaccharides, and cell contents. These components are suggested to be responsible for the beneficial effects of yeast on animal performance (Weedman et al., 2011). In the current study, pigs provided diets with HY40 were heavier at the end of the nursery period versus pigs fed diets without HY40. Previously, pigs fed inactivated yeast at inclusion levels between 0.5% and 1.5% had greater BW regardless of inclusion levels compared to the control, but only when fed at 1.0% and 1.5% was incidence of diarrhea significantly reduced (Boontiam et al., 2020). When inclusion levels were lower (0.01%–0.04%), postbiotic yeast was ineffective in improving growth in growing and finishing pigs (Levesque et al., 2016). Yeast fed as a pro- or prebiotic resulted in inconsistent growth performance responses between studies, some reporting heavier pigs (Liu et al., 2017; Shen et al., 2017) and others reporting no improvement over the control (Weedman et al., 2011; Che et al., 2017; Chance et al., 2021). For example, yeast fed as a probiotic or prebiotic at 0.2% inclusion level had no improvement in growth performance, (Weedman et al., 2011; Che et al., 2017). However, at 0.5%, pigs were heavier at 42 d post-weaning and had greater ADG (20%–25%) than those fed the control diet (Shen et al., 2017). Additional improvements in growth were not observed when inclusion rates were >1.0% (Shen et al., 2017). Feeding yeast in equal parts live yeast, and fermented yeast (0.1% total), with inclusion level halved in phase II, reported no improvements in growth performance (Chance et al., 2021). Yeast processing affects the efficacy of yeast at various inclusion levels; however, improvements in growth performance are more consistent when included in ≥0.5%. Although improvements in growth performance were observed in the current study for pigs provided diets containing HY40, these differences were not seen until the last 14 d of the nursery period, despite inclusion levels being half of that provided in the first 14 d. Further refinement in dose may be required, and perhaps phase I diets require greater inclusion levels than what was supplied in this study.

Supplementing nursery diets with ZnO has been beneficial when included at pharmacological levels (≥2,000 ppm). Previous research has demonstrated that ZnO increases ghrelin resulting in greater feed intake (Yin et al., 2009). Although pigs have nutritional requirements for Zn for various biological processes (i.e., enzyme function, and metabolic processes) at the cellular level (NRC, 2012), pharmacological concentrations of ZnO have been found to increase growth in pigs post-weaning (NRC, 2012). The growth performance results from the present study agreed with a meta-analysis of 26 studies that showed that pharmacological levels of ZnO increased ADG, ADFI, and G:F in post-weaning pigs (Sales, 2013). In these studies, levels of ZnO inclusion ranged from 500 to 5,000 ppm and pigs were fed between 1 and 4 wk post-weaning. Overall, enhanced feed intake appeared to account for a part of the improvement in growth when piglets are fed pharmacological levels of ZnO. However, concerns regarding excess Zn excretion of animals fed pharmacological levels of ZnO is the reason for regulatory pressure to stop this practice (EMA, 2017). Additionally, the current study found that when pigs were fed diets containing high levels of ZnO, Zn excretion was between 5 and 8 times greater than non-ZnO fed pigs, showing that concerns around excess Zn excretion are warranted. Additionally, when pigs were fed HY40 and ZnO in combination, Zn excretion on days 7 and 14 was 20% and 32% greater, respectively, than when ZnO was fed alone. On day 28, however, HY40 + ZnO did not have greater Zn excretion than when ZnO was fed alone. It is possible that at high levels, yeast cell walls are binding to Zn causing the increase in Zn excretion (Gupta and Diwan, 2017). There were limited benefits on growth performance when HY40 and ZnO were fed in combination, aside from greater ADFI during phase II for HY40 + ZnO pigs compared to all other treatments. This did not translate to greater ADG or G:F. With limited improvements in growth and elevated Zn excretion on days 7 and 14, when these were fed in combination at 0.5% and 3,000 ppm for HY40 and ZnO, respectively, feeding them in combination was not beneficial. During phase II when their inclusion levels were halved, there was no additional Zn excreted when fed in combination. Different inclusion levels of HY40 and ZnO should be explored further, and perhaps similar improvements in growth could be achieved at lower inclusion levels of each when fed in combination.

In addition to growth performance, feeding yeast cell wall products have also been shown to improve nutrient digestibility, fecal scores, gut integrity, and immune modulation (Kiarie et al., 2010; Kiarie et al., 2011; Kiarie et al., 2012). In the current study, however, we did not observe an impact attributed to HY40 supplementation on concentration of SCFA, gene expression for nutrient transporters, cytokines, tight junction proteins, or oxidative stress 14 d post-weaning. These previous studies observed improvements in growth performance in conjunction with these other measurements (Kiarie et al., 2010; Kiarie et al., 2011), whereas in the current study, there was no change in growth performance during phase I, when nutrient transporters, oxidative stress, and tight junction proteins were measured. Differences may be apparent at the end of phase II for these parameters since growth performance for pigs fed HY40-containing diets was also improved during this time. Additionally, no differences in fecal consistency scores were observed (as an indicator of post-weaning diarrhea) for the first week post-weaning, and these scores ranged between soft and normal feces indicating generally healthy pigs. In a previous study, feeding yeast-products helped maintain growth after an immune challenge (e.g., mycotoxin contamination), despite not seeing differences in growth performance prior to the challenge (Holandra et al., 2020). Since the data suggest that the pigs used in this study were relatively healthy, it is possible that greater benefits of HY40 would be observed after an immune or stress (e.g., transport) challenge.

As mentioned, yeast supplementation has improved growth of pigs following an immune challenge, which is a result of β-glucans in the yeast. β-glucans found in yeast increase circulating immunoglobulins, particularly secretory IgA (Anwar et al., 2017). The yeast product used in this study contained 40% cell wall materials including β-1,3/1,6 glucans and mannan oligosaccharides. The immune modulatory effects of yeast differ from ZnO such that yeast affects the immune response at a tissue level (i.e., in intestinal mucosal), whereas ZnO has both a local (gut; Kiarie et al., 2011) and systemic effect via the blood (Yin et al., 2009). In this context, we measured concentration of plasma IgA in jejunal tissues and in plasma and observed significant interaction between HY40 and ZnO in plasma IgA concentrations, such that HY40 + ZnO resulted in greater concentrations of circulating IgA than either control or feeding them separately. As expected, HY40 increased IgA concentrations in jejunal tissues versus non-HY40-containing diets. From these results, it is expected that pigs provided HY40 or HY40+ZnO, would be able to perform better when immune challenged. This study was conducted in a research facility and perhaps improvements in growth would be more apparent in a commercial setting where stocking density, and pathogen load is typically greater. In future studies, growth performance and gastrointestinal physiology parameters should continue to be measured later in the nursery phase, and modifications in sanitation procedures could be employed to mimic the immune challenge in commercial farms.

In addition to improvements in feed intake, studies have shown that ZnO improved colonic morphology, mucin composition and mRNA expression of genes related to innate immunity and inflammatory processes, (Liu et al., 2014) intestinal microbiota (Pieper et al., 2012) and attenuation of enterotoxigenic *E. coli* diarrhea in a disease challenge model (Owusu-Asiedu et al., 2003; Kiarie et al., 2008). These effects typically result in an intestinal environment that is favorable to the growth of beneficial bacteria (i.e., relatively high *lactobacilli* [LAB] and low *E. coli* concentrations). Our findings suggest that co-feeding ZnO and HY40 created an environment that limited *E. coli* growth in the ileum, and diets with ZnO resulted in pigs with a greater LAB: *E. coli*. *Escherichia coli* becomes more abundant when the colon has a higher pH, thinner mucosal layer, reduced expression of tight junction proteins, additionally, greater *E. coli* concentrations is related to a greater incidence of diarrhea and reduced weight gain (Pluske, 2016). In the current study, pigs provided diets with ZnO had lower *E. coli* concentrations, greater mRNA expression of nutrient transporters (EAAC1, PepT1, SGLT1), and tight junction protein (OCLN). Although providing HY40 and ZnO in combination had positive effects on *E. coli*, and plasma IgA concentrations 14 d post-weaning, providing these in combination did not result in improved growth over feeding HY40 or ZnO alone, and increased Zn excretion during phase I. Therefore, in areas where pharmacological ZnO is permitted, feeding in combination can be an option, but optimization of their respective inclusion levels would need to be studied further. Although lactic acid accounts for the greater proportion of ileal digesta SCFA (Kiarie et al., 2007), butyric acid is more important for gut health. Butyric acid accounts for most of the energy that is utilized by intestinal epithelial cells, has immunoregulatory effects, and increases mucin production (Anwar et al., 2017); therefore, from our results, pigs provided diets with ZnO had an improved gastrointestinal environment and an improved innate immune system.

With pharmacological levels of ZnO being banned in the EU, producers are looking for sustainable ways to maintain post-weaning growth and reduce the incidence of enteric disease in newly weaned pigs. Maintenance of growth requires a healthy intestinal villus architecture, minimal gut inflammation, and microbiota predominated by beneficial bacteria (Mantis et al, 2011). An effective replacement for high levels of ZnO must have functional roles in the gut that promote a healthy, functional gastrointestinal tract. The current study demonstrated that HY40 had positive effects on growth, histomorphology, nutrient digestibility, and immune activation in nursery pigs. The mechanisms differ with ZnO, but overall growth performance at the end of the nursery period is comparable. In general, however, there were limited additive benefits of providing both ZnO and HY40 to nursery pigs. Therefore, further refinement of dose is required when feeding in combination to ensure economic feasibility, and in areas where pharmacological ZnO inclusion is prohibited, HY40 is an effective replacement.

## Abbreviations

ATTD: apparent total tract digestibility
B^0^AT-1: sodium-dependent neutral amino acid transporter
BW: body weight
CD: crypt depth
CP: crude protein
CW: crypt width
DM: dry matter
DMI: dry matter intake
EAAC-1: excitatory amino-acid carrier 1
GE: gross energy
IgA: Immunoglobulin A
IL-6: Interleukin 6
IL-10: Interleukin 10
N: nitrogen
OCLN: Occludin
qPCR: quantitative real-time polymerase chain reaction
RT-PCR: real-time polymerase chain reaction
SCFA: short-chain fatty acids
SOD-1: superoxide dismutase
VH: villus height
VW: villus width
ZO-1: Zonula occuldens-1

## References

[CIT0001] Adeola, O. 2000. Digestion and balance techniques in pigs. pp. 923–936 in Swine nutrition. Boca Raton (FL): CRC Press.

[CIT0002] Agyekum, A. K., A. Regassa, E. Kiarie, and C. M. Nyachoti. 2016. Nutrient digestibility, digesta volatile fatty acids, and intestinal bacterial profile in growing pigs fed a distillers dried grains with solubles containing diet supplemented with a multi-enzyme cocktail. Anim. Feed Sci. Tech. 212:70–80. doi:10.1016/j.anifeedsci.2015.12.006.

[CIT0003] Anwar, M. I., F. Muhammad, M. M. Awais, and M. Akhtar. 2017. A review of β-glucans as a growth promoter and antibiotic alternative against enteric pathogens in poultry. World. Poultry Sci. J. 73:651–661. doi:10.1017/S0043933917000241.

[CIT0004] AOAC. 2005. Official methods of analysis of AOAC International. Gaithersburg (MD): AOAC International.

[CIT0005] Apajalahti, J., K. Vienola, K. Raatikainen, V. Holder, and C. A. Moran. 2019. Conversion of branched-chain amino acids to corresponding isoacids—an in vitro tool for estimating ruminal protein degradability. Front. Vet. Sci 6:311. doi:10.3389/fvets.2019.00311. 31620454 PMC6759480

[CIT0006] Boontiam, W., C. Wachirapakorn, and P. Phaengphairee. 2020. Effects of hydrolyzed yeast supplementation on growth performance, immunity, antioxidant capacity, and microbial shedding in weaning pigs. Vet. World. 13: 1902–1909. doi:10.14202/vetworld.2020.1902-1909. 33132604 PMC7566246

[CIT0007] Carleton, H. M., R. A. B. Drury, and E. A. Wallington. 1980. Carleton’s histological technique. Oxford, UK: Oxford University Press.

[CIT0008] CCAC. 2009. Guidelines on the care and use of farm animals in research, teaching and testing. Ottawa, Canada: Canadian Council on Animal Care.

[CIT0009] Chance, J. A., J. M. DeRouchey, R. G. Amachawadi, V. Ishengoma, T. G. Nagaraja, R. D. Goodband, J. C. Woodworth, M. D. Tokach, H. I. Calderón, Q. Kang, J. A. Loughmiller, B. Hotze, and J. T. Gebhardt. 2021. Live yeast and yeast extracts with and without pharmacological levels of zinc on nursery pig growth performance and antimicrobial susceptibilities of fecal Escherichia coli. J. Anim. Sci. 99(12): 1–10. doi:10.1093/jas/skab330. PMC8664753 34752618

[CIT0010] Che, L., Q. Xu, C. Wu, Y. Luo, X. Huang, B. Zhang, E. Auclair, T. Kiros, Z. Fang, Y. Lin, et al. 2017. Effects of dietary live yeast supplementation on growth performance, diarrhoea severity, intestinal permeability and immunological parameters of weaned piglets challenged with enterotoxigenic *Escherichia coli* K88. Br. J. Nutr. 118:949–958. doi:10.1017/S0007114517003051. 29166952

[CIT0011] Ciesinski, L., S. Guenther, R. Pieper, M. Kalisch, C. Bednorz, and L. H. Wieler. 2018. High dietary zinc feeding promotes persistence of multi-resistant *E. coli* in the swine gut. PLoS One. 13:e0191660. doi:10.1371/journal.pone.0191660. 29373597 PMC5786291

[CIT0012] Dove, C. R., and L. C. Alworth. 2015. Blood collection from the orbital sinus of swine. Lab Animal. 44:383–384. doi:10.1038/laban.869. 26398611

[CIT0013] EMA. 2017. CVMP. EMA—Zinc Oxide—Annex II—Scientific Conclusions and Grounds for the Refusal of the Marketing Authorization and for Withdrawal of the Existing Marketing Authorizations. Amsterdam, The Netherland: EMEA.

[CIT0014] Eriksen, E. O., E. Kudirkiene, A. E. Christensen, M. V. Agerlin, N. R. Weber, A. Nødtvedt, J. P. Nielsen, K. T. Hartmann, L. Skade, L. E. Larsen, et al. 2021. Post-weaning diarrhea in pigs weaned without medicinal zinc: risk factors, pathogen dynamics, and association to growth rate. Porc. Health Manag. 7:54. doi:10.1186/s40813-021-00232-z. PMC8501929 34627400

[CIT0015] Gupta, P., and B. Diwan. 2017. Bacterial Exopolysaccharide mediated heavy metal removal: a review on biosynthesis, mechanism and remediation strategies. Biotechnol. Rep. 13:58–71. doi:10.1016/j.btre.2016.12.006. PMC5361134 28352564

[CIT0016] Holandra, D. M., A. Yiannikouris, and S. W. Kim. 2020. Investigation of the efficacy of a postbiotic yeast cell wall-based blend on newly-weaned pigs under a dietary challenge of multiple mycotoxins with emphasis on deoxynivalenol. Toxins. 12:1–19. doi:10.3390/toxins12080504. PMC7472238 32781569

[CIT0017] Kakhki, R. A., D. W. Ma, K. Price, J. Moats, N. Karrow, and E. Kiarie. 2020. Enriching ISA brown and Shaver white breeder diets with sources of n-3 polyunsaturated fatty acids increased embryonic utilization of docosahexaenoic acid. Poultry Sci. 99:1038–1051. doi:10.1016/j.psj.2019.09.002. 32036961 PMC7587772

[CIT0018] Kettunen, H., E. van Eerden, K. Lipiński, T. Rinttilä, E. Valkonen, and J. Vuorenmaa. 2017. Dietary resin acid composition as a performance enhancer for broiler chickens. J. Appl. Anim. Nutr. 5:1–9. doi:10.1017/jan.2016.10.

[CIT0019] Kiarie, E., S. Bhandari, D. O. Krause, M. Scott, and C. M. Nyachoti. 2010. Weaned piglet responses to *Escherichia coli* K88+ oral challenge when fed diets containing a *S. cerevisiae* fermentation product with or without in-feed antibiotics. J. Anim. Sci. 88 (Suppl.2):653(Abstr.).

[CIT0020] Kiarie, E., S. Bhandari, M. Scott, D. O. Krause, and C. M. Nyachoti. 2011. Growth performance and gastrointestinal microbial ecology responses of piglets receiving *Saccharomyces cerevisiae* fermentation products after an oral challenge with Escherichia coli (K88). J. Anim. Sci. 89(4). doi:10.2527/jas.2010-3424. 21148775

[CIT0021] Kiarie, E., D. O. Krause, and C. M. Nyachoti. 2008. Net fluid and electrolyte losses in enterotoxigenic Escherichia coli infected piglet small intestine upon perfusion with fumaric acid, zinc oxide, egg yolk antibodies or carbadox. Can. J. Anim. Sci. 88:485–488. doi:10.4141/cjas08011.

[CIT0022] Kiarie, E., C. M. Nyachoti, B. A. Slominski, and G. Blank. 2007. Growth performance, gastrointestinal microbial activity, and nutrient digestibility in early-weaned pigs fed diets containing flaxseed and carbohydrase enzyme. J. Anim. Sci. 85:2982–2993. doi:10.2527/jas.2006-481. 17686904

[CIT0023] Kiarie, E., M. Scott, D. O. Krause, H. Khazanehei, E. Khafipour, and C. M. Nyachoti. 2012. Interactions of *Saccharomyces cerevisiae* fermentation product and in-feed antibiotic on gastrointestinal and immunological responses in piglets challenged with Escherichia coli K88+. J. Anim. Sci. 90(Suppl 4):1–3. doi:10.2527/jas.52977. 23365265

[CIT0024] Kiarie, E., B. A. Slominski, D. O. Krause, and C. M. Nyachoti. 2009. Gastrointestinal ecology response of piglets’ diets containing non-starch polysaccharide hydrolysis products and egg yolk antibodies following an oral challenge with Escherichia coli (k88). Can. J. Anim. Sci. 89(3):341–352. doi:10.4141/cjas09007.

[CIT0025] Kiarie, E., M. C. Walsh, and C. M. Nyachoti. 2016. Performance, digestive function, and mucosal responses to selected feed additives for pigs. J. Anim. Sci. 94(Suppl.3):169–180. doi:10.2527/jas.2015-9835.

[CIT0026] Kisielinski, K., S. Willis, A. Prescher, B. Klosterhalfen, and V. Schumpelick. 2002. A simple new method to calculate small intestine absorptive surface in the rat. Clin. Exp. Med. 2(3):131–135. doi:10.1007/s102380200018. 12447610

[CIT0027] Levesque, C. L., S. Jalukar and J. F. Patience. 2016. Inclusion of a hydrolyzed yeast product in grow/finish pig diets reduced mortality. J. Anim. Sci. 94 (Suppl. 2):71. (Abstr.) doi:10.2527/msasas2016-153.

[CIT0028] Liu, P., R. Pieper, J. Rieger, W. Vahjen, R. Davin, J. Plendl, W. Meyer, and J. Zentek. 2014. Effect of dietary zinc oxide on morphological characteristics, mucin composition and gene expression in the colon of weaned piglets. PLoS One. 9(3):e91091. doi:10.1371/journal.pone.0091091. 24609095 PMC3946750

[CIT0029] Livak, K. J., and T. D. Schmittgen. 2001. Analysis of relative gene expression data using real-time quantitative PCR and the 2^–ΔΔCT^ method. Methods. 25:402–408. doi:10.1006/meth.2001.1262. 11846609

[CIT0030] Mantis, N. J., N. Rol, and B. Corthésy. 2011. Secretory IgA’s complex roles in immunity and mucosal homeostasis in the gut. Mucosal. Immunol. 6:603–611. doi:10.1038/mi.2011.41. PMC3774538 21975936

[CIT0031] Myers, W. D., P. A. Ludden, V. Nayigihugu, and B. W. Hess. 2004. A procedure for the preparation and quantitative analysis of samples for titanium dioxide. J. Anim. Sci. 82:179–183. doi:10.2525/2004.821179x. 14753360

[CIT0032] NRC. 2012. Nutrient requirements of swine. 11th rev. ed. Washington, DC: National Academy of Sciences Press.

[CIT0033] Owusu-Asiedu, A., C. M. Nyachoti, and R. R. Marquardt. 2003. Response of early-weaned pigs to an enterotoxigenic *Escherichia coli* (K88) challenge when fed diets containing spray- dried porcine plasma or pea protein isolate plus egg yolk antibody, zinc oxide, fumaric acid, or antibiotic. J. Anim. Sci. 81:790–798. doi:10.2527/2003.8171790x. 12854816

[CIT0034] Pieper, R., W. Vahjen, K. Neumann, A. G. Van Kessel, and J. Zentek. 2012. Dose-dependent effects of dietary zinc oxide on bacterial communities and metabolic profiles in the ileum of weaned pigs. J. Anim. Physiol. An. N. 96:825–833. doi:10.1111/j.1439-0396.2011.01231.x. 21929727

[CIT0035] Pluske, J. R. 2016. Invited review: aspects of gastrointestinal tract growth and maturation in the pre-and postweaning period of pigs. J. Anim. Sci. 94:399–411. doi:10.2527/jas2015-9767.

[CIT0036] Sales, J. 2013. Effects of pharmacological concentrations of dietary zinc oxide on growth of post-weaning pigs: a meta-analysis. Biol. Trace Elem. Res. 152(3): 343–349. doi:10.1007/s12011-013-9638-3. 23463368

[CIT0037] Salminen, S., M. C. Collado, A. Endo, C. Hill, S. Lebeer, E. M. M. Quigley, M. E. Sander, R. Shamir, J. R. Swann, H. Szajewska, et al. 2021. The International Scientific Association of Probiotics and Prebiotics (ISAPP) consensus statement on the definition and scope of postbiotics. Nat. Rev. Gastroenterol. Hepatol. 18:649–667. doi:10.1038/s41575-021-00440-6. 33948025 PMC8387231

[CIT0038] Shen, Y. B., V. Fellner, I. Yoon, and S. W. Kim. 2017. Effects of dietary supplementation of *Saccharomyces cerevisiae* fermentation product to sows and their offspring on growth and meat quality. Transl. Anim. Sci. 1:45–53. doi:10.2527/tas2016.0005. 32704629 PMC7235506

[CIT0039] Tajadini, M., M. Panjehpour, and S. H. Javanmard. 2014. Comparison of SYBR Green and TaqMan methods in quantitative real-time polymerase chain reaction analysis of four adenosine receptor subtypes. Adv. Biomed. Res. 3:85. doi:10.4103/2277-9175.127998. 24761393 PMC3988599

[CIT0040] Weedman, S. M., M. H. Rostagno, J. A. Patterson, I. Yoon, G. Fitzner, and S. D. Eicher. 2011. Yeast culture supplement during nursing and transport affects immunity and intestinal microbial ecology of weanling pigs. J. Anim. Sci. 89:1908–1921. doi:10.2527/jas.2009-2539. 21606447

[CIT0041] Yin, J., X. Li, D. Li, T. Yue, Q. Fang, J. Ni, X. Zhou, and G. Wu. 2009. Dietary supplementation with zinc oxide stimulates ghrelin secretion from the stomach of young pigs. J. Nutr. Biochem. 20(10):783–790. doi:10.1016/j.jnutbio.2008.07.007. 18926680

